# Editorial: Frontiers in Hemoglobinopathies: New Insights and Methods

**DOI:** 10.3389/fmolb.2021.632916

**Published:** 2021-03-19

**Authors:** Roberta Risoluti, Roshan Colah, Stefano Materazzi

**Affiliations:** ^1^ Department of Chemistry, Sapienza University of Rome, Rome, Italy; ^2^ ICMR - National Institute of Immunohaematology (Rtd), Mumbai, India

**Keywords:** hemoglobinopathies, thalassemia, screening, new methods, advances, rare disease screening

**Editorial on the Research Topic**

**Frontiers in Hemoglobinopathies: New Insights and Methods**

Inherited hemoglobin disorders are estimated to be the most common monogenic diseases worldwide. Deletions or point mutations in *α*- or β-globin genes cause abnormalities in the synthesis or in the structure of hemoglobin, leading to *α* and β thalassaemia syndromes or structural hemoglobin variants, respectively. The highest prevalence of both diseases has been recorded in the Mediterranean area, Middle East, Indian subcontinent, Southeast Asia and north coast of Africa, moreover, as a result of migration of ethnic minority groups with high frequency of these mutations, the hemoglobin disorders are also present in northern European and American countries.

Sickle cell anemia (SCA) is by far the most common hemoglobin disorder (Ware et al., 2017), followed by the serious forms of thalassemia syndromes including also the co-inheritance of β thalassaemia with hemoglobin E and hemoglobin S (β-thalassemia major, HbE/β-thalassemia, HbS/thalassemia, Hb Bart hydrops, HbH disease) (Weatherall, 2010). Considerable research has been carried out in the last decades to broaden our knowledge both on clinical and therapeutic aspects of haemoglobinopathies, to improve the laboratory diagnosis and to introduce diagnostic tools useful for screening programs in areas with a high incidence of these diseases. The aim of this Research Topic is to provide an update on hemoglobinopathies and this ebook collects important and significant reports covering several areas. Advances in laboratory diagnosis are proposed by new diagnostic methods and screening tools for prevention programs and differential diagnosis of anemia. Advances in clinical management and treatment of thalassemia major, thalassemia intermedia and sickle cell anemia are also reported. Diagnosis of hemoglobinopathies requires several laboratory tests (Green et al., 2015) and in this book is reported a review (Munkongdee et al.) that highlights the primary biochemical and molecular techniques commonly used in the clinical lab today, including red blood cell indices (Risoluti et al., 2018), hemoglobin analysis by HPLC and capillary electrophoresis and several molecular technologies for analysis of *α* and β globin gene mutations.(Nadkarni et al., 2019). The progress in molecular diagnostics has also provided new methods to define the globin gene mutations and correlate genotype to phenotype. A recently applied molecular analysis for the screening of thalassemias is the Next Generation Sequencing (NGS) that has provided interesting preliminary data of an increase in accurate diagnoses and new understanding of these diseases. The main limitation of using NGS techniques is the very high cost for screening purposes. The development of effective and inexpensive techniques for thalassemia and hemoglobin variant screening is extremely important, especially in countries that have populations with a high percentage of these disorders (Risoluti et al., 2016; Colah et al., 2018). The application of thermogravimetry (De Angelis Curtis et al., 2008; Materazzi and Risoluti, 2014; Risoluti et al., 2017) coupled to chemometrics (Massart et al., 1998; Materazzi et al., 2017) as a new screening method to perform an early diagnosis of thalassemia and sickle cell disease has been proposed (Risoluti et al.)., providing an innovative multilevel test for Hemoglobinopathies that simultaneously identifies and classifies Sickle Cell Disease from Thalassemia. The novel test is able to simultaneously perform a simple and fast diagnosis of sickle cell anemia or thalassemia, in a single analysis of few microliters of non-pretreated whole blood at low cost, and with high accuracy. This innovative multilevel test has been applied for diagnosis of a case of congenital hemolytic anemia (Risoluti et al.) for which the common first level diagnostic tests were not able to find the erythrocyte congenital defect. The test evidenced the presence of a hemoglobinopathy and the molecular analysis confirmed the presence of a rare hemoglobin variant (Risoluti et al.). National haemoglobinopaties screening programmes are performed in the areas with high frequencies of these diseases and a “one-stop” screening protocol for hemoglobinopathy traits and iron deficiency has been proposed for the detection of carriers of HbE- β-thalassemia and iron deficiency in Sri Lanka (Allen et al.). Infections are major complications and a common cause of mortality and morbidity in thalassemia, being severe anemia, splenectomy, iron overload, and immune abnormalities (Sabbieti et al., 2005) predisposing factors to infections. Moreover, transfusion associated viral infections, especially hepatitis C increase the risk of liver disease leading to liver fibrosis, cirrhosis, and hepatocellular carcinoma. The development of the new effective Direct-acting Antiviral Agents (DAAS) toward the HCV infection is discussed (Maffei et al.) and the HCV eradication followed to DAAS treatment seems to improve the iron overload demonstrating a synergic action between DAAS therapy and iron chelation. Other complications of iron overload are the endocrine comorbidities including hypogonadism, hypothyroidism, diabetes mellitus, and bone diseases. Studies regarding adrenal impairment are heterogeneous in terms of methods used, incidence and populations studied. Hematological and hormonal data of a large group of thalassemia patients associated with their clinical history have been evaluated and showed a prevalence of adrenal insufficiency in thalassemia patients, particularly in male subjects (Poggi et al.). The authors pointed out the limit of the methods usually used to investigate adrenal insufficiency in adult thalassemia patients and highlighted the importance of specific, accurate and validated methods to achieve a real diagnosis. The increase in life expectancy of thalassemia patients has also changed their perspective including their desire to have children. A retrospective study (Sorrentino et al.) describes the experience of pregnancies in women affected by thalassemia major, thalassemia intermedia and sickle cell anemia by applying well-defined protocols that cover all the critical aspects of pregnancy in these diseases. The pregnancies have been followed by a multidisciplinary team from the preconception phase until the post-partum period, with a close monitoring of the maternal and fetal conditions in order to reduce complications and improve their outcomes. A particular focus was placed on SCA and related vascular complications. The hemorheological profile of SCA subjects have been studied (Caprari et al.) and high blood viscosity, increased RBC aggregation, and decreased RBC deformability have been demonstrated. These impaired flow properties were associated with RBC membrane protein oxidation, with degradation of spectrin and increased membrane-bound globin. The comparison between SCA patients with and without transfusion dependence showed metabolic and structural RBC oxidative damage which was significantly different. The hypothesis that gender could play a role in determining the clinical course of SCA was investigated (Ceglie et al.) by analyzing the clinical records of 39 pediatric patients with a diagnosis of SCD (hemoglobin SS genotype) and focusing on gender differences in various aspects of the disease comprising both acute symptoms and late complications. Gender-related differences were found in pain crisis frequency and severe infectious and cardiovascular complications which were significantly increased in the male population. The development and treatment of pain, in particular, neuropathic pain in sickle cell disease patients is poorly understood and impedes our progress toward the development of novel therapies to treat pain associated with sickle cell disease. The orexin/hypocretin system offers a novel approach to treat chronic pain and hyperalgesia (Richardson et al.). Neuronal activation differences in the orexin system as a result of neuropathic pain testing have been studied in a mouse model of sickle cell disease. Identifying specific orexin neuronal populations that are integral in neuropathic pain processing will allow us to elucidate mechanisms that provide a more selective, targeted approach in treating of neuropathic pain in sickle cell disease. Microfluidic technology in sickle cell research has been reviewed by Aich et al. as a valuable tool for a biophysical characterization of sickle red cells, to measure their deformability under defined oxygen gradient and shear, and to test *in vitro* models of intercellular interaction on endothelialized or adhesion molecule-functionalized channels. These studies will allow to understand adhesion in sickle microenvironment, to characterize biomechanics and microrheology, and to develop diagnostic technologies. In conclusion the papers in this e-book collect contributions of different expertises in the field of hematology and analytical methods and will address different specific problems such as novel diagnostic methods, prevention programmes, screening tools and prediction of biomolecules for diagnosis, adding new insights and perspectives in the Hemoglobinopathies.
